# The Effectiveness of the Barton’s Intervention Program on Reading Comprehension and Reading Attitude of Students with Dyslexia

**Published:** 2011

**Authors:** Zeinab Mihandoost, Habibah Elias

**Affiliations:** 1 Department of Educational Studies, University Putra Malaysia, 43400 UPM Serdang, Selangor Darul Ehsan, Malaysia

**Keywords:** Attitude, Comprehension, Dyslexia, Intervention program

## Abstract

**Objective: **The current research tested the differences in reading attitude and reading comprehension in the dyslexic students between the control group and the experimental group following the Barton intervention program.

**Methods:** Dyslexia screening instrument and reading text were employed in order to identify dyslexic students. The population of the study included 138 dyslexic students studying in schools in Ilam, Iran. From this population, 64 students were randomly selected and assigned to an experimental group as well as a control group. The experimental group was taught for 36 sessions, using the Barton’s method at two levels, and ten lessons were provided to improve the reading skill. The reading comprehension and reading attitude instruments were employed for the measurement of the attitude and comprehension before and after the intervention program.

**Results:** The analysis of covariance showed a significant difference between the control group and the experimental group following the Barton intervention program.

**Conclusion:** This study showed that dyslexic students learned to read, and a more direct instruction related to decoding could influence their progress more than the general exposure to education.

## Introduction

Dyslexia is one of the ordinary learning disorders around the world. It is a common disorder among any population but it is more common in elementary schools (1), where one out of 10 students might suffer from severe reading problems in comparison with the normal intelligence and high-quality educational chance. Recently, psychologists and neuroscientists reported that dyslexia had the same origin in different languages: reduced activity in the left temporal parietal cortex (2). Researchers have started to study the ways in which influential factors impact the subsequent development of reading skills in students with dyslexia. They found that the important factors are reading attitude and reading comprehension (3, 4).

Attitude toward reading plays an essential role in the development and use of reading skills. Richeck, List and Lerner (5) stated that “the final success of education is strongly affected by the reader’s attitude”. Furthermore, Lipson and Wixson (6) concluded that “the student’s attitude toward reading is an essential factor affecting reading performance”. The result of the studies by Polychroni (7), Lazarus and Callahen (8) showed that students diagnosed with reading disability had negative attitudes toward reading. Attitudes can also consist of one’s affinity for a particular activity. The importance of the affective characteristics of learning-disabled students has long been noted, and these students have often negative characteristics. Despite this somewhat general acceptance in the field (9), it has not been definitively ascertained whether the negative affective variables cause learning disability, they are its consequences, they are related in origin to the actual disability, or they are simply behaviors which just happen concurrently with the difficulty in learning. Nevertheless, there seems to be a general agreement that the prolonged failure experiences in the learning skill of the disabled children have a profound and lasting effect (10).

Students’ attitudes toward reading are positively linked to reading improvement. When students pay attention to what is being taught and they have access to materials that interest them, learning and attitudes improve (11). Reading attitude is typically viewed as a multidimensional concept related to the functions of reading. A number of models of attitudes toward reading have been proposed (12, 13). In all models, the decision to read is viewed to be determined largely by attitudes toward reading. Mathewson (13) supported attitude’s function as a causal agent in the reading process. The factors that may influence children’s positive attitudes toward reading are what the child believes about others’ expectations, and what the child believes about his or her reading outcome and the type of prior reading experience. Children’s prior beliefs and cognitive-affective knowledge may affect their reading comprehension (14).

Comparisons with low-skilled and without disability students suggest that students with learning disability have negative attitudes toward reading (7,15). Studies have documented that students with dyslexia who received reading instruction in special education and resource rooms expressed attitudes toward academic and recreational reading that equaled or exceeded those expressed by low and average students without disability, implying that perceptions of ability are important (8). Moreover, when individuals with dyslexia get involved in voluntary reading in the areas of personal interests, their reading ability improves (16).

Students with dyslexia are inactive readers and lack comprehension strategies that would assist them in understanding the meaning of the text (17). These students have difficulty summarizing and comprehending the text because they tend to reflect on the information that is not central and tend to omit pertinent pieces of information (18). In particular, one of the largest problems for dyslexic students is differentiating the peripheral details from the main ideas in the text (19). As noted by Daneman (20), vocabulary is partially an outcome of comprehension skills, and likewise, reading comprehension is partially an outcome of vocabulary. Dyslexic students need specific strategy training in monitoring comprehension and specific strategy instruction in previewing and activating prior knowledge (21), predicting (22), clarifying and summarizing in order to facilitate their understanding of the content (23).

Comprehension of reading is an energetic procedure that needs a planned and thoughtful interaction between the reader and the text. As the readers try to comprehend the material they read, they must bridge the gap between the information presented in the written text and the knowledge they possess. Thus, reading comprehension involves thinking. The reader’s background knowledge, interest and the reading situation affect the comprehension of the material. Each person’s integration of the new information in the text with what is already known will yield unique information (24). All reading instructions should be provided for the development of reading comprehension. Reading comprehension is a major problem for dyslexic students. Comprehension skills do not automatically evolve after word-recognition skills have been learned. Although most dyslexic students eventually learn the basics of word-recognition skills, many of them continue to have great difficulty with tasks that require comprehension of complex passages. These students need to learn strategies that will help them become active readers who can understand the text (25).

The important strategy to help the dyslexic students by means of the multisensory techniques is the Barton program. The Barton program is an Orton-Gillingham program which simultaneously influences the multisensory, explicit, and systematic phonics programs created by Susan Barton. This program is a one-to-one tutoring system that will greatly improve the reading skills of the students who suffer from learning disability (26).

This study aims to compare the experimental and control groups of dyslexic students before and after the Barton intervention program. The research questions are as follows:

1. Does the Barton intervention program improve the reading attitude of the dyslexic students?

- Does the Barton intervention program improve the recreational reading of dyslexic students?
- Does the Barton intervention program improve the academic reading of dyslexic students?

1. Does the Barton treatment program improve the dyslexic students’ reading comprehension?

This study is guided by the subsequent research hypotheses:

1. There is a statistically significant difference in reading attitude between the control group and the experimental group of the students with dyslexia after the Barton intervention program.

- There is a statistically significant difference in recreational reading between the control group and experimental group of the students with dyslexia after the Barton intervention program.
- There is a statistically significant difference in academic reading between the control group and experimental group of the students with dyslexia after the Barton intervention program.

1. There is a statistically significant difference in reading comprehension between the control group and the experimental group of the students with dyslexia after the Barton treatment program.

## Materials and Methods

*Procedure*

In this study, the students of fourth and fifth grades with dyslexia were identified by using a questionnaire called “Dyslexia Screening Instrument”. Two 100-word passages with 10 comprehension questions from the students’ book were selected and were assigned to the students to read. Their marks were also scrutinized in the first semester and it was found that their marks were lower than those of the students without dyslexia in the reading skills. To examine their IQ, Raven’s test was performed, and the students with an average IQ of higher than 90 made up the population of this research. Finally, 138 dyslexic students in the fourth and fifth grades were selected. The population of dyslexic students consisted of 40 male and 38 female fifth graders, and 37 male and 22 female fourth graders. Their age ranged from 10 to 12. The researcher used the table of random numbers to select 64 dyslexic students and assigned them to a control group and an experimental group, each with 32 students. “Reading Attitude” and “Reading Comprehension” scales were conducted in both groups. The students were orally directed how to complete the Attitude toward Reading Scale (27) and Reading Comprehension (28). The researcher read the items aloud and observed whether the students understand the instrument, and provided assistance when it was necessary. The demographic variables such as age, gender, and IQ were obtained as well. When the students had completed answering the questionnaire (approximately 30 minutes later), they returned to their classroom.

*Intervention*

Barton intervention program (26) was used in this study. Barton Reading and Spelling System includes ten levels. Each level is broken into lessons and each lesson, in turn, is further broken into procedures. In this study, only first and second levels are taught with some adjustments. Considering the fact that in the Persian language, there are 26 consonants and 6 vowels, 6 lessons were specified for level two. Like Barton’s program (26), in the adjustment program, teaching procedures started with the easy level and gradually became difficult. Since the instruction tools were not available in Persian, the researcher provided the necessary tools based on Barton’s Program. The instruction tools included: 1) color coded letter tiles, 2) word lists, 3) cards on which one word was written in blue consonants and red vowels 4) whiteboard, 5) blue and red markers, and 6) a notebook for dictation along with red and blue pencils, erasers, and sharpeners. According to Barton’s program (26), level one was taught first. Then, 6 consonants, and one vowel were taught in each session of level two. Sometimes, due to the difficulty of some consonants or vowels, some lessons were repeated for 2 or 4 sessions. Therefore, one by one instruction was done for 36 sessions in 12 weeks, and each week with three sessions and each session lasting 45 minutes. It seems necessary to note that the students received the intervention in their school. The instruction time was set by the tutors. If the students could not learn a lesson properly, the lesson would be repeated until she/he learned it.

*Pilot study*

The purpose of the pilot study was to evaluate the appropriateness of the use of the instruments. For the pilot study, 30 dyslexic students with similar characteristics from Ilam schools were randomly selected. The students consisted of 19 males and 11 females. This study was carried out from 1^st^ March to 5^th^ March, 2010. Then, the data were entered into SPSS version 18 software to determine the reliability of the scales. The reliability test was applied by calculating the Cronbach’s alpha on the variables to measure the inter-item reliability. There was consistency in the following variables: Reading attitude and reading comprehension. Internal consistency is generally measured by Cronbach’s alpha, a value calculated from the pair-wise correlation between items. Internal consistency ranges between zero and one. Cronbach’s alpha coefficient of reliability and alpha of 0.70 are normally considered to indicate a reliable set of items (29). Cronbach’s alpha reliabilities of the Reading Attitude and Reading comprehension were 0.79 and 0.83 respectively. The results of the reliability coefficient showed that there was a high reliability for these instruments. Thus, these instruments were considered appropriate to be employed in this study.

*Validity*

In this study, to ascertain the validity of Reading Attitude and Reading Comprehension Scales, 10 psychology experts graded the scales from 1 to 5. The acceptable degree figures are shown in table 1. Although there is no statistics for content validity, in Table 1 a statistical figure, namely mean was introduced. It should be stated that what has been put forward in table 1 is the acceptability degree criteria determined by the judges.

**Table 1:** Mean judges rank

Judges	Attitude	Comprehension
1	4.4	4.85
2	4.4	4.7
3	4.45	4.75
4	4.5	4.92
5	4.5	4.78
6	4.4	4.9
7	4.15	4.7
8	4.35	4.8
9	4.2	4.8
10	4.3	4.87

Table 1 the rank given by 10 psychology experts for reading attitude and reading comprehension scales, based on Gregory (30), and Cohen (31), and

*Measures*

Five instruments were utilized in this research as follows: 1) the Dyslexia Screening Instrument (DSI), 2) Reading Text, 3) Passage Comprehension Scale 4) Reading Attitude and 5) Raven’s Progressive Matrices.

*Reading Attitude:*

In 1990, Mckenna & Kear defined that the Elementary Reading Attitude Survey (ERAS) is a 20-item questionnaire that asks students to rate their attitudes toward reading. Each item presents a brief, simply worded statement about reading, followed by four pictures of the comic strip character, Garfield the cat in varying pictorial poses. The percentile ranks can be obtained for two component subscales: recreational reading attitude and academic reading attitude. The recreational items focus on reading for fun outside the school setting and the academic subscale examines the school environment and reading schoolbooks. A total reading attitude percentile rank can also be computed as an additive composite of the recreational and academic scores (27). Cronbach’s alpha, a value developed mainly to determine the internal consistency of attitude scales (32) was calculated at each grade level for both subscales and for the composite score. These coefficients ranged from 0.74 to 0.89 (27). The validity of the academic subscale was tested by examining the relationship of the scores to the reading ability. The educators classified norm-group students as having a low, average, or high overall reading ability. The mean subscale scores of the high ability readers (M=27.7) significantly exceeded the mean of low ability readers (M=27<0.001), confirming that the scores showed how the students actually felt about reading for academic purposes. In this research, the scores on the scale had an acceptable reliability (Attitude=0.75).

*Passage Comprehension:*

The initial Passage Comprehension items involve symbolic learning, or the ability to match a rebus (pictographic representation of a word) with an actual picture of the object. The subsequent items are in a multiple-choice plan and need the student to point to the picture represented by a phrase. The remaining items require the students to read a short passage and identify a missing keyword that makes sense in the context of that passage. The items become increasingly difficult by removing pictorial stimuli and by increasing the length of the passage, the level of vocabulary and the complexity of the syntactic and semantic cues. In this adapted cloze process, the subject must apply a variety of comprehension and vocabulary skills. Performance on this reading task can be compared directly with the performance in one of the counterpart oral comprehension tasks. The passage comprehension has a median reliability of 0.83 in the age ranging between 5 to 9 years, and 0.88 in the adult age range (28). In this study, the Cronbach’s alpha reliability for the scale was 0.85, while the test-retest reliability was 0.87.

*Dyslexia Screening Instrument (DSI):*

Dyslexia Screening Instrument (DSI) consists of checklists of basic neuropsychological skills designed by Coon, Waguespack, and Polk in 1994. This instrument is a rating scale designed to describe the cluster characteristics associated with dyslexia and to discriminate between the students who display the cluster characteristics and the students who do not. It is designed to measure “entire population of the students who exhibit reading, spelling, writing, or language-processing difficulties” (33). The DSI is designed to be used by grade 1 through 12 students (age 6 through 21). The Internal consistency reliability coefficients (i.e. 0.99) for elementary students were determined using Cronbach’s coefficient alpha; and the inter rater reliability for elementary students was 0.86 that was assessed by determining the homogeneity of the statements and consistency of ratings across the examiners. Coon *et al* (1994) stated that “content was based on an extensive review of the relevant literature and on the experts in the field of dyslexia” (P.20). Construct validity was supported by the discriminate analysis classifications which placed elementary and secondary students accurately (98.2% and 98.6% respectively). A classroom teacher who has worked directly with a student for at least four months should complete the DSI scale. This will result in a rating that will be more accurate because the teacher has observed the student over a lengthy period of time and can compare the performance with that of his classmates. For an elementary student, the preferred rater is the teacher who instructs the student in a variety of subjects. The teacher should complete the DSI form (based on the questionnaire answer: Never exhibits, Seldom exhibits, Sometimes exhibits, Often exhibits and Always exhibits). In this study, Cronbach’s alpha reliability of the scale was 0.89.

*Raven’s Progressive Matrices test:*

Raven’s Standard progressive Matrices (RSPM) test was constructed to measure the educative component of g (general IQ) as defined in Spearman’s theory of cognitive ability (34). Kaplan and Saccuzzo (35) stated that “research supports the RSPM as a measure of general intelligence. The advanced form of the matrices contains 48 items, presented as one set of 12 (set I), and another of 36 (set II). Items are again presented in black ink on a white background, and become increasingly difficult as progress is made through each set. These items are appropriate for 5-65 years of age. Lynn and Vanhanen (36) summarized a number of studies based on normative data for the test collected in 61 countries. The internal consistency reliability estimate for the Raven progressive Matrics total raw score was 0.85 in the standardization sample of 929 individuals. This reliability estimate for the revised RSPM indicates that the total raw score on the RSPM possesses “good” internal consistency reliability as provided in the guidelines of the U.S Department of Labor (37) for interpreting a reliability coefficient. The RSPM has been widely used for decades as a measure of educative ability “the ability to evolve high level constructs which make it easier to think about complex situations and events” (38). In an extensive analysis of the cognitive processes that distinguish between higher scoring and lower scoring examinees on the standard progressive matrices and advanced progressive matrices, Carpenter, Just and Shall (39) described the Raven’s test “a classic test of analytic intelligence”. In this research, Cronbach’s alpha reliability of the scale was 0.83.

*Reading text:*

The reading texts were developed by the researcher based on the content of the fourth and fifth grade textbooks. As during the administration of the research 80 percent of the book had been taught, the developed test was based on 80 percent of the Persian textbooks. The tests were evaluated by the fourth and fifth grade teachers and they were approved after 3 times of revision. The test included a story with one hundred relevant words understandable to each educational level, followed by 10 questions which indicated the students’ level of understanding. The students were required to read out the test aloud and answer the questions. To determine reliability, Cronbach’s alpha was employed. The reliability coefficients for reading tests for the fourth and fifth grades were 0.87 and 0.90 respectively.

*Statistical analysis*

The analysis of covariance (ANCOVA) was employed in the current study. Because ANCOVA includes a covariate in the model, it can help reduce the residual variation. The basic Analysis of Covariance Design is a just pretest-posttest randomized experimental design; that is, the pretest measure is the same one as the posttest measure. The pre-program assessment does not have to be a pretest. It can be any variable assessed prior to the intervention program. It is also possible for a research project to have more than one covariate. The pretest is occasionally called a “covariate” because of the way it is used in the data analysis (40). The SPSS (version 18) was utilized for the analysis of the data. P-value<0.05 was considered statistically significant.

## Results

The results of the study are presented in two parts: descriptive results and the results related to the hypotheses. In Table 2, means and standard deviations are shown for both the experimental group and the control group. The results relevant to the research hypotheses are shown in Tables 2 and 3.

**Table 2:** Mean and standard deviation for reading attitude (subscales) and reading motivation

	Experimental Group			Control Group		
Test	M	SD	N	M	SD	N
Attitude	67.51	5.02	31	48.06	12.25	30
Recreation	35.45	3.38	31	24.63	5.64	30
Academic	32.06	2.81	31	23.43	6.87	30
Comprehension	34.35	7.29	31	27.66	9.12	30

Table 2 shows that the means in reading attitude, recreational reading, academic reading, and reading comprehension in the control group are lower than those in the experimental group.

**Table 3:** Analyses of covariance for reading attitude (subscale) and reading comprehension

Test	F	df	ρ	Mean Square	Sum of Squares
Attitude	65.56	1	.000	5734.36	5734.36
Recreation	82.26	1	.000	1777.87	1777.87
Academic	40.96	1	.000	1127.69	1127.69
Comprehension	39.15	1	.000	657.06	657.06

Table 3 shows that a one-way ANCOVA where pre-test reading attitude, recreational reading, academic reading and reading comprehension were co-varied; the main effect of the treatment on post-test reading attitude, recreational reading, academic reading and reading comprehension was significant (F=65.56, ρ=.000; F=82.26, ρ=.000; F=40.96, ρ=.000; F= 39.15, ρ=.000).

Means score for pretest and posttest reading attitude. The mean score for reading attitude in the pretest is lower than the posttest in the Standard Reading Attitude (27).

**Figure 1:**
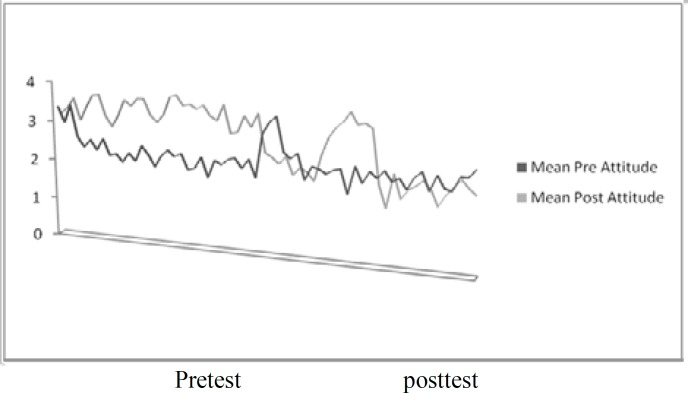
Pre- and post-intervention reading attitude

## Discussion

The current research aimed to investigate the effect of the Barton intervention program on the reading attitude and reading comprehension of students with dyslexia studying at fourth and fifth grades in Ilam, Iran, in the academic year 2010. The first research hypothesis stated that there could be a statistically significant difference in attitude of the students with dyslexia in the control group and the experimental group after the Barton Intervention Program. The first research hypothesis was confirmed at ρ<.000. The results of the study showed that the intervention program was effective in increasing the attitude towards reading in students with dyslexia. The results of this study were in line with researches (17, 26, 41) that showed intervention programs increased the academic skills of students with dyslexia. Such studies showed that attitude was an important factor in academic achievement.

According to Berliner (42), achievement is influenced by attitude as well as ability. “It is a well-known psychological principle that attitude influences a person’s choice of activities as well as effort and persistence at tasks” (p.126). Alexander and Filler (43) identified several variables that seem to be associated with attitudes toward reading. Some of these variables are achievement, the teacher, the classroom and special programs. As the teachers attempt to improve the students’ attitudes toward reading, they should keep these ideas in mind. That is to say, they should have a positive feeling toward the students. The students need to be commended for their efforts. The teacher’s awareness of student’s attitudes toward reading is essential. A student’s attitude toward reading materials affects the comprehension of those materials. Teachers should be well-informed that students’ attitudes toward reading are formed by parents and their home environment.

Studies show that reading attitude is affected by academic achievement. According to Johnson (44), attitudes toward reading are possibly formed as a result of success achievement or failure with the task of reading. Though students with good reading ability may have positive attitudes toward reading, the students who are poor readers often have to overcome negative reading attitudes in order to improve their reading skills. In order to clarify the finding of the study, it can be mentioned that since the intervention program results in academic achievement of dyslexic students, the participation of the dyslexic students on a one-to-one basis in the intervention program would increase the individual capabilities of this group of students.

The second hypothesis was investigated and showed that there was a statistically significant difference in the reading comprehension between the control group and the experimental group of the students with dyslexia after the Barton intervention program. The results confirmed that there was a significant difference in the reading comprehension of the experimental group, who received the treatment. Apparently, the results were in line with several studies done in this area (26, -). Likewise, these studies revealed that the intervention program improved the reading comprehension. Notably, dyslexic students needed direct instructions of alphabet, since teaching alphabet directly makes teaching of primary reading easier. Besides, the studies showed that if multi-sensory methods were used in teaching the dyslexic students, their level of learning would increase (26, 49). Multi-sensory methods, such as the Barton intervention program, can improve the dyslexic children’s reading comprehension. The results of the recent studies on the importance of reading comprehension specify that intervention programs are specifically significant in teaching dyslexic students to acquire reading comprehension. Thus, teachers can use this program to improve dyslexic students’ reading comprehension.

## Notes

**Declaration of Interest:** None.

## References

[1] NeuroDys (2008). German Dyslexia and Dyscalculia Association (BVL) International Conference-State-of-the- Art in Dyslexia and Dyscalculia Research. http://www.neurodys.com.

[2] Paulesu E, Demonet JF, Fazio F, McCrory E, Chanoine V, Brunswick N (2001). Dyslexia: Cultural diversity and biological unity. Science.

[3] Good C V (1973). Dictionary of education.

[4] Williams JE (1994). Gender differences in high school students' efficacy-expectation/ performance discrepancies across four subject matter domains. Psychol Sch.

[5] Richeck MA, List LK, Lerner J (1989). Increasing the achievement of your remedial reading students.

[6] Lipson MY, Wixson KK (1992). Assessment and instruction of reading disability an interactive approach.

[7] Polychroni F, Koukoura K, Anagnostou I (2006). Academic self-concept, reading attitude and approaches to learning of children with dyslexia: do they differ from their peers?. Eur J Spec Needs Educ.

[8] Lazarus DB, Callahan T (2000). Attitudes toward Reading Expressed by Elementary School Students Diagnosed with Learning Disabilities. J Reading Psychol.

[9] Rogers H, Saklofske DH (1985). Self-concepts, locus of control and performance. Expectations of Learning Disabled J Learn Disabil.

[10] Bryan TS, Pearl R (1979). Self-concept and locus of control of learning disability students. J Clin Child Psychol.

[11] Hidi S (1990). Interest and its contribution as a mental resource for learning. Rev Educ Res.

[12] Greaney V, Neuman SB (1990). The functions of reading: a cross-cultural perspective. Read Res Quart.

[13] Mathewson G, Ruddell RB, Ruddell MR, Singer H (1994). Model of attitude influence upon reading and learning. Theoretical model and process of reding. (p.1131-1161).

[14] Ruddell RB, Unrau NJ, R. B. ruddell, M.R. ruddell, H. singer (1994). Reading as a meaning construction process: The reader, the text, and the teacher. theoretical models and processes of reading.

[15] van Kraayenoord CE, Schneider WE (1999). Reading achievement, metacognition, self-concept and interest: A study of German students. Eur J Psychol Educ.

[16] Fink R (1995). successful dyslexics: a constructivist study of passionate interest reading. J Adolesc Adult Literacy.

[17] Torgesen JK, p.McCardle, V. Chhabra (2004). Lessons learned from research on intervention for students who have difficulty learning to read. The voice of evidence in reading research (PP.

[18] Winograd PN (1984). Strategic difficulties in summarizing texts.

[19] Wong BYL (1991). Learning about learning disabilities.

[20] Daneman M, R. Barr, M.L. Kamil, P. Mosenthal, P.D. Pearson (1991). Individual differences in reading skills. Handbook of reading research.

[21] Langer JA (1984). Literacy instruction in american schools: Problems and perspectives. Ame J Edu.

[22] Readence JE, Bean TW, Baldwin RS (1998). Content area reading: An integrated approach.

[23] Torgesen JK, M. Snowling, C. Hulme Recent discoveries from research on remedial intervention for students with dyslexia. Presentations and Publications.

[24] National Reading Panel (2000). Education students to read: An evidence based assessment of the scientific research literature on reading.

[25] Lerner J (2006). Learning disability and related disorder, characteristic and teaching strategy.

[26] Barton S (2000). Intervention program, an Orton influenced multisensory structured.

[27] McKenna MC, Kear DJ (1990). Measuring attitude toward reading: A new tool for teachers. The Reading Teacher.

[28] Woodcock RW, Mather N, Schrank FA (2004). Diagnostic Reading Battery.

[29] De vaus D (2002). Analysing social science data.

[30] Gregory RJ (2004). Psychology teesting-hestory, principle, and application.

[31] Cohen RL (2005). Exercises in psychological testing and measurement, an introduction to tests and assessment.

[32] Cronbach L J (1951). Coefficient alpha and the internal structure of tests. Psychometrika.

[33] Coon B, Waguespack M, Pollk J (1994). Instrument for screening of dyslexia students.

[34] Raven JRJC, Court JH (1998). Raven manual: Section 1, general overview.

[35] Kaplan RM, Saccuzzo DP (1997). Psychological testing: Principle, application and issue.

[36] Lynn R, Vanhanen (2002). Intelligence and the wealth of nations.

[37] U. S. Department of Education (2002). Guidance for the Reading Earliest Program.

[38] Raven J, Raven C, Court H (1998). Higher progressive matrices.

[39] Carpenter PA, Just MA, Shell P (1990). What one intelligence test measures: a theoretical account of the processing in the Raven progressive matrices test. Psychol Rev.

[40] Trochim WM (2006). The research methods knowledge base. http://www.socialresearchmethods.net/kb/.

[41] Davis RD (1994). The talent of dyslexia.

[42] Gage L, Berliner C (1998). Psycholoy for teaching.

[43] Alexander JE, Filler RC (1976). Attitudes and reading.

[44] Johnson DS (1981). Naturally acquired learned help-lessness: the relation ship of school failure to achievement behavior, attributions, and self-concept. J Edu Psychol.

[45] Carnine D, Silbert J, Kame'enui EJ (1990). Direct instruction in reading.

[46] DeFord D, Ellis W (1991). On nobel thoughts, or toward a clarification of theory and practice within a whole language framework. All Language and creation of literacy.

[47] Snow C, Burns M, Griffin P (1998). Report of the committee on the prevention of reading disability in youth children.

[48] Rivers KO, Lombardino LJ (1998). Generalization of early metalinguistic skill in a phonological decoding study with first grads at risk for reading disability. Int J Lang Comm Dis.

[49] Orton J (1976). A guide to teaching phonics.

